# Cyclooxygenase inhibitors impair CD4 T cell immunity and exacerbate *Mycobacterium tuberculosis* infection in aerosol-challenged mice

**DOI:** 10.1038/s42003-019-0530-3

**Published:** 2019-08-05

**Authors:** Rasmus Mortensen, Helena Strand Clemmensen, Joshua S. Woodworth, Marie Louise Therkelsen, Tehmina Mustafa, Kristian Tonby, Synne Jenum, Else Marie Agger, Anne Ma Dyrhol-Riise, Peter Andersen

**Affiliations:** 1 Department of Infectious Disease Immunology, Statens Serum Institut, 2300 Copenhagen S, Denmark; 2 Centre for International Health, Department of Global Public Health and Primary Care, University of Bergen & Department of Thoracic Medicine, Haukeland University Hospital, 5021 Bergen, Norway; 3 Department of Infectious Diseases, Oslo University Hospital, 0424 Oslo, Norway; 4 Institute of Clinical Medicine, University of Oslo, 0424 Oslo, Norway; 5 Department of Clinical Science, University of Bergen, 5020 Bergen, Norway; 6 Department of Immunology and Microbiology, University of Copenhagen, 2200 Copenhagen N, Denmark

**Keywords:** Tuberculosis, Tuberculosis, Bacteria

## Abstract

Tuberculosis, caused by infection with *Mycobacterium tuberculosis* (Mtb), kills over 1.6 million people each year despite availability of antibiotics. The increase in drug resistant Mtb strains is a major public health emergency and host-directed therapy as adjunct to antibiotic treatment has gained increased interest. Cyclooxygenase inhibitors (COXi) are frequently used drugs to alleviate tuberculosis related symptoms. Mouse studies of acute intravenous Mtb infection have suggested a potential benefit of COXi for host-directed therapy. Here we show that COXi treatment (ibuprofen and celecoxib) is detrimental to Mtb control in different mouse models of respiratory infection. This effect links to impairments of the Type-1 helper (Th1) T-cell response as CD4 T-cells in COXi-treated animals have significantly decreased Th1 differentiation, reduced IFNγ expression and decreased protective capacity upon adoptive transfer. If confirmed in clinical trials, these findings could have major impact on global health and question the use of COXi for host-directed therapy.

## Abstract (web summary)

Rasmus Mortensen et al. examine the effects of cyclooxygenase inhibitors in an aerosol *Mycobacterium tuberculosis* infection mouse model. They find that the treatment diminish bacterial control and impairs adaptive immunity by reducing the differentiation of CD4 T-cells into Type 1 T helper cells.

## Introduction

Tuberculosis (TB), caused by *Mycobacterium tuberculosis* (Mtb), constitutes one of the biggest global health problems of our time. An estimated 10 million new cases and 1.6 million deaths in 2017 makes TB the leading infectious cause of death and more than one fourth of the world’s population are latently infected^1^. Even for drug sensitive TB, the global cure rate is <85% and the availability of effective therapy is further threatened by the growing epidemic of multi-drug resistant Mtb strains.

Host-directed therapy, i.e. modulation of specific host immune pathways, including those that impact inflammation and immunopathology, constitutes an attractive avenue with potential for treatment of both drug sensitive and multi-drug resistant TB. Among the already approved medicines, non-steroidal anti-inflammatory drugs (NSAIDs), including cyclooxygenase inhibitors (COXi), have been proposed as candidates for host-directed therapy due to their potential to reduce inflammation and hereby resolve host-meditated immune pathology^2–4^. In addition, COXi are also commonly used drugs to alleviate TB related symptoms.

COXi target either one or both isoforms of the COX enzyme (COX-1 and COX-2), which are involved in the synthesis of prostaglandins, most notably PGE2 that is a potent immune modulator^5^. During the establishment of Mtb infection, PGE2 appears to be essential for bacterial control, possibly by promoting apoptosis of infected macrophages and counteracting production of type I interferons, whereas elevated PGE2 levels at later stages seems to promote disease progression^6–9^. Initial animal studies using COXi in aerosol-infected Balb/c mice showed diverging results; there was no observed effect of ibuprofen or acetylsalicylic acid (aspirin) as stand-alone treatment, but aspirin was found to antagonize antibiotic treatment with isoniazid whereas ibuprofen/aspirin enhanced pyrazinamide treatment^10,11^. However, more recent studies have convincingly shown that treatment with ibuprofen or aspirin alone reduces the bacterial burden and prolongs survival after intravenous (i.v.) infection of highly TB-susceptible C3HeB/FeJ mice^12–14^. In this model, mice rapidly developed severe lung pathology and inflammation, which was reduced by COXi^13^. While this model reflects some aspects of human disease (e.g. development of necrotic/hypoxic lesions)^15,16^, we speculated whether this beneficial influence was consistent in other less pathology prone animal models mimicking respiratory Mtb infection. The aim of the present study was therefore to investigate the effect of COXi in a standardized low-dose aerosol infection model, where mice acquire a stable chronic infection that is CD4 T cell dependent^17^. Not only does aerosol infection more accurately reflect the natural respiratory infection route in humans, but establishment of a stable infection also allows investigation of long-term treatment effects.

In this study, we show for the first time that treatment with COXi (celecoxib and ibuprofen) can increase the bacterial burden after aerosol infection. When the infection route is changed from aerosol to i.v., COXi treatment decreases inflammation and reduces the bacterial burden in the lung, as previously reported. In the aerosol model, there is little or no impact on inflammation or recruitment of innate cells. Instead, COXi treatment impairs formation of immune memory, reduces Type 1 helper T cell (Th1) function/differentiation and diminishes the protective capacity of CD4 T cells. Given that COXi, and NSAIDs in general, are some of the most used drugs in clinical practice, this should be further investigated. If COXi pose a risk of TB progression in Mtb infected subjects, it could have major impact on the global TB epidemic and question the relevance of these drugs as candidates for host-directed therapy.

## Results

### COXi treatment exacerbates Mtb aerosol infection in CB6F1 mice

**Fig. 1:**
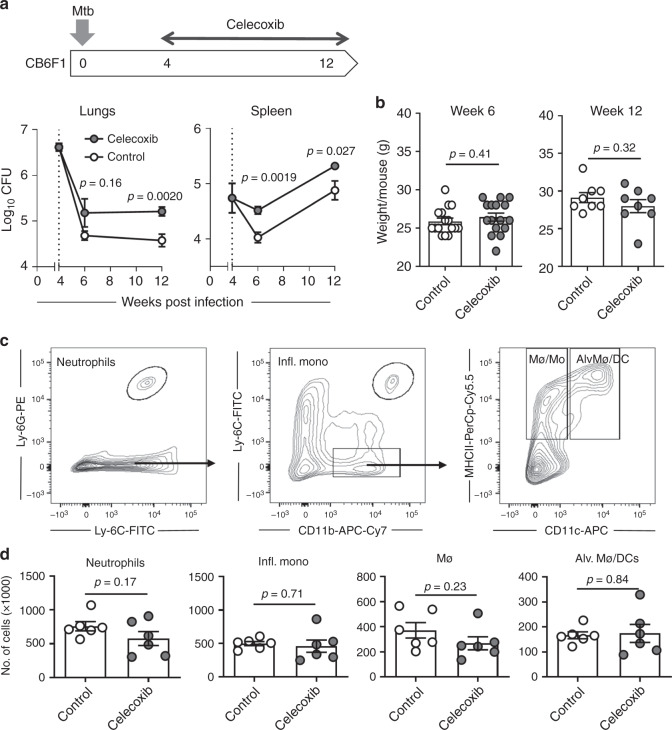
Celecoxib treatment exacerbates Mtb aerosol infection. **a** Female CB6F1 mice of 5–8 weeks of age were infected with ~25–50 CFU Mtb Erdman by the aerosol route (week 4 (*n* = 4), week 6 (*n* = 6), week 12 (*n* = 8)). 4 weeks into the infection, mice were fed with normal chow or chow containing 500 ppm celecoxib. Mtb bacilli were enumerated by plating lung and spleen homogenates 2 and 8 weeks later (week 6 and 12 of the infection). **b** Animal weight was monitored at week 6 (*n* = 16) and week 12 (*n* = 8) to ensure that the observed differences were due to treatment effects and not because of weight-loss from altered feeding habits. **c** Two weeks after treatment (week 6 of the infection), the pulmonary influx of myeloid-derived cells was determined by staining lung cells for CD11b, CD11c, Ly-6C, Ly-6G, and MCHII. Neutrophils were defined as CD11b^+^/Ly-6C^dim^/Ly-6G^+^, inflammatory monocytes as CD11b^+^/Ly-6C^high^/Ly-6G^−^. Gating on Ly-6C^−^/Ly-6G^−^CD11b^+^ cells, macrophages/monocytes (Mø/Mo) were defined as CD11b^+^/MHCII^+^/CD11c^−^ and a mixed population of alveolar macrophages/dendritic cells (Alv. Mø/DCs) was defined as CD11b^+^/MHCII^+^/CD11c^+^. **d** Individual mice were analyzed for the number of neutrophils, inflammatory monocytes, macrophages and alveolar macrophages/dendritic cells in the lung 2 weeks into the treatment (*n = *6). Bars represent mean ± SEM and *p* values were calculated with an unpaired two-tailed *t*-test comparing treated and non-treated animals

Previous publications have demonstrated that COXi treatment during Mtb infection can improve outcome and reduce bacterial burden^12–14^. In the present study, we investigated this in more detail using a standardized low dose TB aerosol model in CB6F1 mice. We initially evaluated the effects of celecoxib, a specific COX-2 inhibitor that can be formulated efficiently into the animals’ chow^18^. This circumvents the need to perform daily oral administrations and eliminates the bias that treated animals are stressed by extensive handling. Mice were challenged with ~25–50 CFU Mtb Erdman and put on chow containing 500 ppm celecoxib after 4 weeks (peak of infection). We found that celecoxib-treated mice had higher bacillary burdens in lungs and spleen after both 2 and 8 weeks of treatment, corresponding to week 6 and 12 of the infection, respectively (Fig. 1a). Importantly, there was no difference in animal weight between the two groups, indicating that addition of celecoxib to the chow did not affect feeding habits (Fig. 1b). We further investigated soluble proinflammatory cytokines in plasma and lung homogenates (IL-1β, KC/GRO, TNFα, IL-6, IL-12p70, and IFNγ) as well as pulmonary influx of myeloid-derived cells 2 weeks into COXi treatment (week 6 of infection). In previous studies, COXi reduced inflammation and neutrophilic infiltration^12–14^, but we found no difference in the number of neutrophils, inflammatory monocytes, macrophages/monocytes or alveolar macrophages/dendritic cells (DCs) in the lungs (Fig. 1c, d and Supplementary Fig. 1). There was a tendency that the expression of MHCII and CD86 was higher on CD11b^+^CD11c^+^ cells in the celecoxib-treated animals, which might be explained by the higher bacterial load (Supplementary Fig. 2A). In general, cytokine levels were low, and there were no differences between the two groups on any of the cytokines that were investigated in both plasma and lung homogenates (Supplementary Fig. 2B, C).

In summary, using our standardized aerosol Mtb infection model in CB6F1 mice, we found that celecoxib treatment increased bacterial burden. This was not associated with differences in pulmonary inflammation or infiltration of innate immune cells, suggesting that the treatment effect was not linked to dysregulation of the innate inflammatory response.

### The COXi treatment effect is dependent on route of infection

**Fig. 2:**
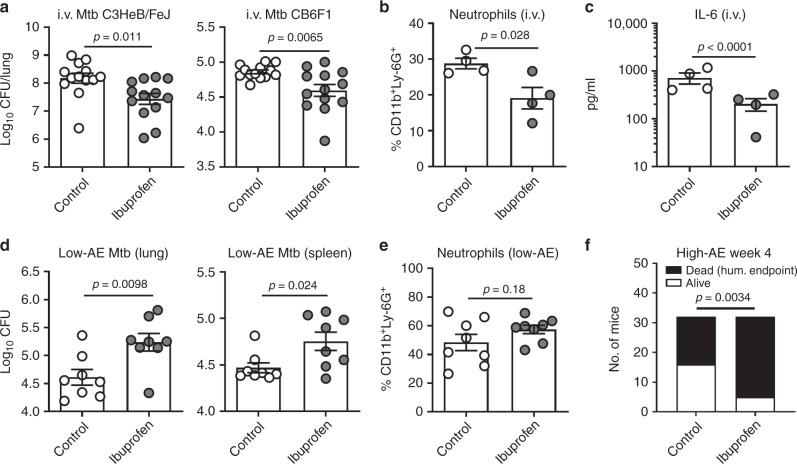
The COXi treatment effect is dependent on route of infection, not a specific COX1/2 inhibitor. **a** Female C3HeB/FeJ or CB6F1 mice of 6 to 8-weeks of age were infected by intravenous (i.v.) injection of 5 × 10^4^ Mtb H37Rv or Erdman respectively. After 3 weeks, the animals were put on a 1-week course of 80 mg/day oral ibuprofen before enumerating Mtb bacilli in the lungs (*n* = 13). **b** The frequency of neutrophils in the lungs of C3HeB/FeJ mice (*n* = 4) was determined by flow cytometry gating on CD11b^+^/Ly-6G^+^ cells 1 week after treatment. **c** Soluble IL-6 was measured along with other pro-inflammatory cytokines (Supplementary Fig. 3) by multiplex analysis of lung homogenates of C3HeB/FeJ mice after 1 week of treatment (*n* = 4). For this analysis, *q* values (adjusted *p* values) were calculated with an ANOVA followed by the Benjamini-Hochberg method to control the false discovery rate during multiple comparisons. **d** 6 to 8-week-old female CB6F1 mice were infected with a low dose of ~25–50 CFU Mtb Erdman by the aerosol (AE) route and put on a course of 80 mg/day oral ibuprofen at week 4 of the infection (as in Fig. 1). After 8 weeks of treatment (week 12 of the infection), Mtb bacilli were enumerated in both lungs and spleen by plating organ homogenates (*n* = 8). Data is shown from one out of two independent experimentswith *n* = 8 and *n* = 6, respectively. **e** Two weeks into the treatment (week 6 of the infection) the frequency of neutrophils in the lungs was determined as before (data from one representative experiment, *n* = 8). **f** 6 to 8-week-old female CB6F1 mice were infected with a high-dose of ~150–300 CFU Mtb Erdman by the aerosol (AE) route and put on a course of 80 mg/day oral ibuprofen at week 3 of the infection. Mice were monitored individually for 1 week and euthanized when they reached pre-defined humane endpoints. The *p* value was calculated using a chi-square test. Bars represent mean ± SEM and *p* values were calculated with an unpaired two-tailed *t*-test comparing treated and non-treated animals (unless otherwise indicated)

Given the unexpected increased bacterial growth after celecoxib treatment in our first experiment, we assessed the robustness of the observation. As previous studies utilized the non-selective COXi, ibuprofen and aspirin, in an i.v. Mtb infection model, we initiated a series of experiments to test whether the treatment outcome was dependent on infection route and/or drug. First, we carefully reproduced the data of Vilaplana et al.^12^ by showing that a 1-week course of 80 mg/day oral ibuprofen lowered the pulmonary bacterial burden after Mtb H37Rv i.v. infection in the highly susceptible C3HeB/FeJ mice (Fig. 2a, left). Secondly, we demonstrated that this effect could be extrapolated to a more Mtb-resistant mouse strain, as the same result was obtained with CB6F1 mice infected i.v. with Mtb Erdman (Fig. 2a, right). Interestingly, and in contrast to the aerosol/celecoxib infection model, ibuprofen treatment after i.v. infection significantly decreased lung infiltration of neutrophils as well as IL-6 (Fig. 2b, c and Supplementary Fig. 3, *p* = 0.028 and *p* < 0.0001).

Having confirmed that ibuprofen had a positive effect in the i.v. infection model, we tested whether this also was the case in the aerosol infection model. For this, we used an experimental design that mirrored the study in Fig. 1, but replaced celecoxib with ibuprofen. CB6F1 mice were infected via the aerosol route and 4 weeks into the infection, they were put on a course of 80 mg/day oral ibuprofen. 8 weeks after treatment (week 12 of the infection), animals were sacrificed and Mtb bacilli were enumerated by plating organ homogenates. Similar to the first experiment with celecoxib, we found that ibuprofen significantly increased the bacterial burden in both the lungs and the spleen (Fig. 2d, *p* = 0.0098 and *p* = 0.024). Consistent with the celecoxib-treatment data (Fig. 1), ibuprofen had no impact on the infiltration of neutrophils (Fig. 2e). Finally, to explore whether the treatment outcome was dependent on bacterial load, we challenged CB6F1 mice with a high-dose Mtb Erdman infection by the aerosol route. Mice were put on oral ibuprofen at week 3 and monitored for 1 week using a validated clinical scoring system (see materials and methods). At week 4, 27/32 animals in the ibuprofen group met the pre-defined humane endpoints for euthanization compared to 16/32 in the control group (Fig. 2f). Together, this indicated that the negative treatment outcome of COXi was dependent on infection route (rather than bacterial load in the model).

In summary, we showed that the negative impact of COXi on Mtb control after aerosol infection was experimentally robust, as the same results were obtained with both celecoxib and ibuprofen as well as after both low- and high-dose Mtb challenge. The effect of COXi treatment appears to depend on the infection model, as ibuprofen treatment decreased inflammation and bacterial burden when the infection was initiated via the i.v. route.

### Celecoxib impairs immune memory and affects CD4 T cell phenotype

**Fig. 3:**
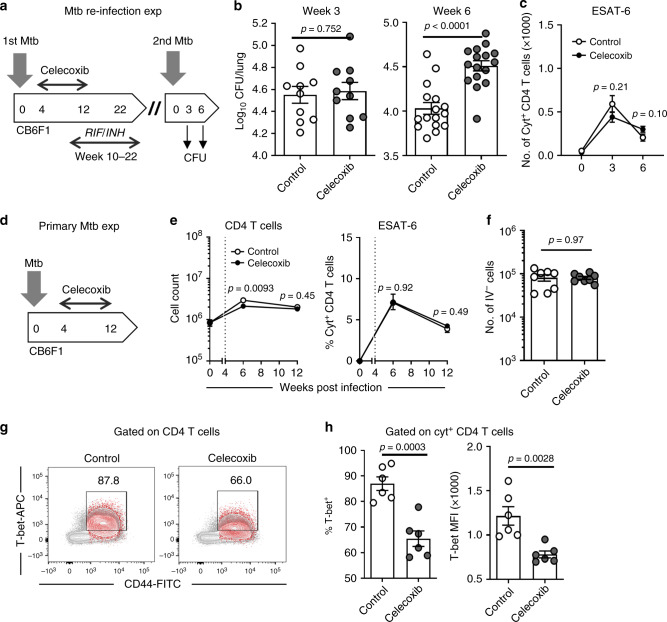
Celecoxib impairs immune memory and affects CD4 T cell phenotype. **a** 6 to 8-week-old female CB6F1 mice were infected with ~25–50 CFU Mtb Erdman by the aerosol route and fed with celecoxib chow for 8 weeks as previously (week 4–12). At week 10, mice were put on antibiotic treatment with rifampicin/isoniazid for 12 weeks to clear the infection (week 10–22). After a 2-week rest to wash out the antibiotics, mice re-infected with 50–100 CFU Mtb Erdman by the aerosol route. **b** Mtb bacilli were enumerated in the lungs at week 3 (*n* = 10) and 6 (*n* = 16) of the re-infection by plating organ homogenates. **c** The number of cytokine positive CD4 T cells (producing any combination of IFNγ, TNFα or IL-2, see and Supplementary Fig. 5) was determined by ICS of lung cells stimulated with overlapping ESAT-6 peptides (*n* = 6). **d** Female CB6F1 mice were infected with ~25–50 CFU Mtb Erdman by the aerosol route and fed with celecoxib chow as previously (same experiment as in Fig. 1). **e** The total number of CD4 T cells as well as the frequency of cytokine producing CD4 T cells (IFNγ, TNFα or IL-2) was determined by ICS of lung cells stimulated with overlapping ESAT-6 peptides (week 0 *n* = 2, week 6 *n* = 6, week 12 *n* = 8). **f** At week 12, the ability of CD4 T cells to home to infected tissue was assessed by enumerating the CD4 T cells that were not stained by i.v. injection of a labeled CD45 antibody (IV^−^). **g** The Th1 differentiation status of activated CD4 T cells (CD44hi) was assessed in both cytokine producing (red) and non-producing (grey) T cells by staining for the linage-specific transcription factor, T-bet (data show a concatenation of every animal in each group). **h** (left) the frequency of T-bet^+^ CD4 T cells in the cytokine producing population of each animal, (right) T-bet expression measured by mean fluorescence intensity (MFI) of the population of T-bet^+^ CD4 T cells (*n* = 6). Bars represent mean ± SEM and *p* values were calculated with an unpaired two-tailed *t*-test comparing treated and non-treated animals

Given that treatment with celecoxib and ibuprofen decreased host resistance following aerosol infection, without affecting inflammation/recruitment of innate cells, we wanted to determine whether the effect was long-lived. For this, we applied an Mtb re-infection model, where CB6F1 mice were treated with celecoxib for 8 weeks as previously followed by a 12-week antibiotic course of isoniazid/rifabutin. Subsequently, the mice were rested for 2 weeks prior to aerosol Mtb re-infection (Fig. 3a). Mice that were treated with celecoxib during the initial infection had significantly increased bacterial burdens in lungs (Fig. 3b) and spleen (Supplementary Fig. 4A) when their second infection progressed from week 3 to week 6, indicating that the COXi-effect was long-lasting and independent on ongoing celecoxib treatment (*p *< 0.0001 and *p* = 0.0015, respectively). There was a slight tendency that the mice in the celecoxib group also had larger changes in histopathology at week 6, but this was not statistically significant (Supplementary Fig. 4B, *p* = 0.28). As there is ample evidence that COXi and/or PGE2 concentrations regulate inducible nitric oxide synthase (iNOS) in macrophages^6,19–21^, we investigated whether the increased bacterial burden in the celecoxib group was associated with compromised iNOS expression. Contrary to the expectation, stained tissue sections of lungs revealed that iNOS expression was elevated in the celecoxib group, correlating with bacterial burden, which suggests that the treatment did not affect antigen-driven iNOS expression (Supplementary Fig. 4C). We then examined whether the initial COXi treatment affected the CD4 T cell recall response and found that the number of Mtb-specific CD4 T cells measured by intracellular cytokine staining (ICS) was not significantly different between groups at any time point (Fig. 3c, *p* = 0.21 and *p* = 0.10).

The impaired control of re-infection in mice treated with celecoxib during the initial infection suggested the involvement of an altered adaptive immune response. Given that the magnitude of the CD4 T cell recall response was unaltered during re-infection, we hypothesized that celecoxib treatment induced changes in the phenotype/quality of the T cell response. To test this, we analyzed the CD4 T cell response in animals receiving celecoxib treatment during the first infection (Fig. 3d). There was a small, but detectable, decrease in the number of CD4 T cells in the lungs of celecoxib-treated mice at week 6 (Fig. 3e) but this difference was gone by week 12. Throughout the infection, there was no difference in the frequency of ESAT-6 responsive CD4 T cells, indicating that T cell priming and/or maintenance was not obstructed by the celecoxib treatment (Fig. 3e). Combinatorial analysis of the cytokine producing CD4 T cells also revealed that the cytokine expression profile was identical in treated and untreated animals (Supplementary Fig. 4D).

Recent literature from murine studies supports that CD4 T cells expressing KLRG1 are less efficient at homing to the infected tissue, which is associated with a less protective T cell phenotype^22–24^. We therefore explored whether the negative effect of celecoxib treatment was associated with KLRG1 expression and/or impaired CD4 T cell homing, but found no differences on either parameter (Fig. 3f, Supplementary Fig. 4E and Supplementary Fig. 5). Finally, since PGE2 is known to influence Th1 differentiation^25^, we investigated whether celecoxib treatment altered cellular expression of the Th1-fate regulating transcription factor, T-bet (Fig. 3g). Indeed, when we analyzed the CD44hi T cell population (grey) and the cytokine producing T cells after Mtb antigen stimulation (red), it was evident that celecoxib treatment significantly decreased both the frequency of T-bet expressing CD4 T cells as well as the T-bet expression level (Fig. 3g, h, *p* = 0.0030 and *p* = 0.0028, respectively).

In summary, we demonstrated that celecoxib treatment during Mtb infection of CB6F1 mice impaired formation of memory immunity with little or no influence on the magnitude of the CD4 T cell response. Instead, we observed a significant drop in the expression of T-bet, indicating that COXi influence Th1 differentiation and/or effector-function of CD4 T cells.

### Celecoxib diminishes the protective capacity of CD4 T cells

**Fig. 4:**
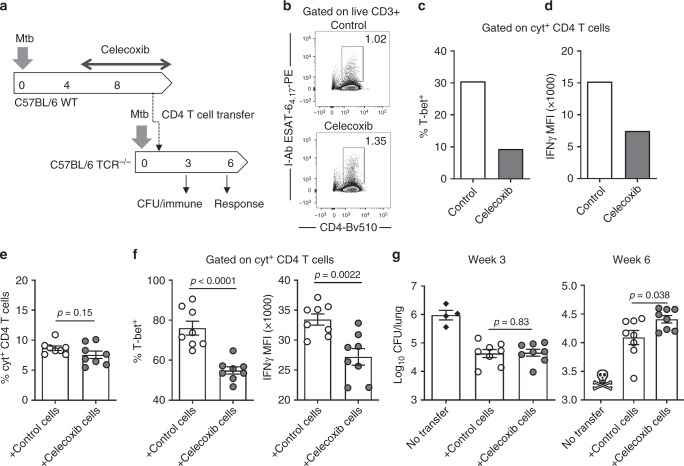
Celecoxib reduce IFNγ expression and diminish the protective capacity of CD4 T cells. **a** WT C57BL/6 donor mice were infected with ~25–50 CFU Mtb Erdman by the aerosol route and treated for 4 weeks with celecoxib (week 4–8) before purifying CD4 T cells from pooled spleen and tracheobronchial/mediastinal lymph nodes. A total of 9.5 × 10^6^ purified CD4 T cells/mouse were transferred into B6.129S2-Tcra^tm1Mom^ recipients (TCR^−/−^) that were infected 7 days prior to cell transfer. **b** At the day of transfer, the pool of purified donor cells were characterized for the frequency of antigen specific CD4 T cells by ESAT-6_4-17_ tetramer staining as well as expression of T-bet (**c**) and IFNγ (**d**) in CD4 T cells producing any combination of IFNγ, TNFα or IL-2 after stimulation with overlapping ESAT-6 peptides. **e** Five weeks after cell transfer (week 6 of the infection), the frequency of cytokine producing CD4 T cells in the lungs of the recipient mice was determined by ICS after ESAT-6 peptide stimulation (*n* = 8). **f** The cytokine producing CD4 T cells were further characterized based on the expression of T-bet and IFNγ. **g** Mtb bacilli were enumerated in the recipient animals at week 3 and 6 of the infection by plating lung homogenates (*n* = 8). All the TCR^−/−^ mice in the control group that did not receive any CD4 T cells met the humane endpoints for euthanasia before week 6. Bars represent mean ± SEM and *p* values were calculated with an unpaired two-tailed *t*-test comparing treated and non-treated animals

To investigate whether the celecoxib-mediated decrease in Th1 differentiation was associated with reduced effector function and/or protective capacity, we performed an adoptive transfer study with cells from either celecoxib-treated animals or untreated control animals (Fig. 4a). Wild type C57BL/6 donor mice were aerosol infected with Mtb and treated for 4 weeks with celecoxib before CD4 T cell isolation from spleen and lymph nodes (LNs; tracheobronchial and mediastinal). At this time point, the frequency of Mtb antigen specific CD4 T cells in the two groups was similar and the T-bet expression was decreased in the celecoxib animals, as previously observed (Fig. 4b, c). This was associated with a decreased IFNγ expression (Fig. 4d). We also analyzed expression of FoxP3 (Tregs), RORγt (Th17) and GATA3 (Th2) and found no effect on any these markers, indicating that the celecoxib treatment specifically affected Th1 cells in our model (Supplementary Fig. 6A, B).

After characterizing the donor cells, 9.5 × 10^6^ CD4 T cells/mouse were transferred into C57BL/6 TCRα^−/−^ recipients that were infected 7 days prior to cell transfer. Six weeks into the infection there was no significant difference in the frequency of cytokine producing CD4 T cells between recipient groups (Fig. 4e, *p* = 0.15). However, the phenotype of decreased T-bet expression was intact post transfer, which was associated with significantly reduced IFNγ expression of the CD4 T cells (Fig. 4f, *p* = 0.0022). Finally, we enumerated Mtb bacilli in recipient mice. Similar to the re-infection experiment (Fig. 3b), we found that mice receiving CD4 T cells from celecoxib-treated animals had a higher lung bacterial burden at week 6 of the infection, indicating that the long-term protective capacity of the CD4 T cells was compromised by the celecoxib treatment (Fig. 4g). Together with the previous experiments, this formally demonstrates that COXi treatment during Mtb aerosol infection can influence Th1 differentiation and/or effector-function of CD4 T cells and diminish their protective capacity.

## Discussion

The outcome of Mtb infection is highly dependent on the inflammatory balance mediated by innate and adaptive components of the immune response and host-directed therapy are exploring strategies to both suppress and augment immune functions^3,26^. COXi are commonly used to treat TB related symptoms, but have also been suggested as host-directed therapy due to their immune-modulating properties^2,4^. Recent murine studies support that COXi during Mtb infection can alleviate excessive inflammation and limit neutrophil infiltration resulting in improved outcome^12–14^. Notably, these studies assessed COXi treatment after i.v. infection of highly susceptible C3HeB/FeJ mice, whereas the primary goal of the present study was to examine the effects of COXi treatment in a standardized low dose aerosol infection model in the less Mtb susceptible CB6F1 mice. Contrary to our expectations, we found that animals treated with celecoxib or ibuprofen had increased bacterial burden compared to untreated control animals. After high-dose aerosol challenge, ibuprofen decreased survival. We hypothesized that the discrepancy between our result and the previous studies could be due to different infection routes and indeed, we observed that ibuprofen treatment lowered the bacterial burden when administered after i.v. infection. In agreement with the previous studies, ibuprofen decreased pro-inflammatory cytokines and neutrophil infiltration after i.v. infection, whereas we observed no effect of COXi on these parameters after aerosol infection. The discrepancy between the results of the aerosol and i.v. models could be caused by several differences in the experimental infection approach. One of the major differences being that the inoculum is over 100–1000 times higher in the i.v. model compared to the aerosol model. Although the resulting bacterial burden in the lung eventually reaches the same level in the two models^27^, delivery of high bacterial numbers directly into the blood stream would cause immediate dissemination to immune-competent organs, like the spleen, resulting in rapid systemic inflammation. In contrast, in the aerosol model only a few bacteria are delivered to the lungs, where they are taken up by alveolar macrophages, which have a less inflammatory profile compared to monocytes and monocyte-derived macrophages that predominate other organs^28–30^. Therefore, speculating that the accelerated systemic inflammation in the i.v. model would favor a beneficial role of anti-inflammatory treatment compared to the aerosol model seems reasonable. In addition to infection route, other factors such as mouse strain, Mtb strain and/or dosage of the COXi drug could also have an impact on the outcome. Indeed, two previous studies have shown that aspirin and ibuprofen had no effect on bacterial burden as stand-alone treatment in aerosol infected BALB/c mice, whereas they had variable effects in enhancing/antagonizing treatment with pyrazinamide and isoniazide^10,11^.

As the innate inflammatory response was not affected by COXi treatment in the aerosol model, we hypothesized that the increased bacterial burden in treated animals could be associated with changes in adaptive immunity. In support of this, we showed that COXi-treated mice were more susceptible to re-infection compared to untreated mice, indicating that memory immunity was impaired by the treatment. Recent mouse studies have focused on lung-homing as a key aspect of adaptive immunity towards Mtb^22–24^, but no effect on the frequency of lung-homing CD4 T cells as determined by in vivo intravascular staining was observed by COXi treatment in the present study. Instead, there was a substantial decrease in T-bet expression of CD4 T cells, which was associated with a drop in IFNγ production. It is well known that cyclooxygenases and their metabolites, in particular PGE2, regulate a multitude of functions in T cell differentiation and effector function^25,31^. Several studies have demonstrated that micromolar-range concentrations of PGE_2_ inhibit Th1 differentiation via downregulation of the IL-12 signaling pathway^32–34^, but other studies have shown that PGE2, in combination with TNFα, promote Th1-inducing dendritic cells and that nanomolar concentrations of PGE2 potentiate Th1 differentiation through downstream signaling of the PGE2 receptor 4 (EP4)^35,36^. In our studies, we observed that COXi treatment decreased Th1 differentiation with little or no impact on IL-12 production, which is most consistent with a model where PGE2 favors Th1 differentiation. Whether the observed treatment effect on Th1 differentiation and effector-function was caused by changes in the downstream signaling pathway of EP4, or if other environmental mechanisms were responsible, should be the focus of future mechanistic studies. We did not perform a detailed analysis of the CD8 T cells and more work is needed to investigate the potential influence of celecoxib on this subset.

T-bet expression of CD4 T cells plays a key role for the development and maintenance of Th1 mediated protection against Mtb and mice deficient in T-bet as well as IFNγ are highly susceptible to Mtb infection^37,38^. However, more recently it has also been demonstrated that T-bet^hi^ Th1 cells are less protective than T cells with intermediate T-bet expression and that CD4 T cell derived IFNγ plays a less pronounced role against pulmonary Mtb infection^39,40^. Hence, in the final experiment, we investigated whether the observed reduction in T-bet expression after COXi treatment was associated with a decreased protective capacity of the CD4 T cell population. We observed that CD4 T cells from celecoxib-treated donors produced less IFNγ than CD4 T cells from untreated control animals and that they were less efficient at controlling bacterial replication when transferred to infected TCRα^−/−^ recipients. Altogether, this shows that COXi treatment can impede adaptive immunity against Mtb, possibly by manipulating the Th1 function of CD4 T cells.

To our knowledge, this is the first demonstration that COXi can affect adaptive immune responses negatively in the context of Mtb infection. Our data do not rule out that other mechanisms also may play a role in our model and that COXi treatment during active TB can alleviate excessive inflammation when present, which is in line with decreased bacterial burden in COXi-treated mice after i.v. infection reported herein and by others^12–14^. Instead, we state that our data sheds light on a potential unidentified risk of COXi treatment.

Although translating findings in mouse models to humans comes with multiple limitations, the frequent use of NSAID to alleviate TB symptoms calls for confirmatory studies in humans. There are no studies exploring the effects of NSAIDs on cure/relapse during active TB, but a few observational studies have investigated a possible association between the use of NSAIDs and development of active TB. Two old case reports and a small retrospective case-control study express a concern for risk of progression to TB in patients using NSAIDs although these provide weak, if any evidence for this concern^41–43^. However, a recent population-wide retrospective case-control study from Taiwan suggested an association between NSAID prescriptions and increased risk of TB, but was unable to demonstrate an association for COXi specifically in the adjusted analysis^44^. This study was corroborated by another Taiwanese study focusing on patients with psoriasis^45^, but a similar association was not found in a Canadian study of patients with rheumatoid arthritis^46^. Although some of these studies express concern that NSAIDs increase the risk of developing TB, retrospective case-control studies are associated with several limitations. Most importantly, they are unable to demonstrate causality and do not control for protopathic bias (e.g. NSAIDs were prescribed to alleviate symptoms of undiagnosed/subclinical TB prior to diagnosis of active TB). In the future, it would therefore be essential to establish whether there is a causative relationship between the use of NSAIDs/COXi and progression to active TB, and subsequently to investigate in more detail how altered eicosanoid balances influence innate as well as adaptive immune responses. Currently, there are registered three clinical trials exploring the effect of NSAIDs on different aspects of active TB (NCT02060006, NCT02781909 and NCT02503839).

In conclusion, we have identified a potential new risk that treatment with COXi during Mtb infection impairs CD4 T cell mediated immunity by decreasing Th1 differentiation and effector function. While it is evident how this could play a role in latent TB infection, where adaptive immunity is responsible for controlling the infection, it is less clear for active TB, where adaptive immunity has already failed and antibiotic therapy restricts bacterial replication. We suggest careful investigation of the impact of COXi on both active and latent TB in diverse animal models as well as clinical trials.

## Materials and methods

### Mice

Five to eight-week-old female C3HeB/FeJ (The ‘Jackson’ Laboratories, Bar Harbor, ME), CB6F1 hybrid mice (female BALB/c × male C57BL/6, ‘Envigo’ Laboratories, The Netherlands), C57BL/6 (Charles River Laboratories, Wilmington, MA) and B6.129S2-*Tcra*^tm1Mom^/J (Jackson), were purchased and randomly assigned to cages at the animal facility at Statens Serum Institut (SSI) upon arrival. Animals were allowed to rest for 1 week before any experimental manipulation and they were allowed free access to water and food throughout the experiment. Mice were weighed throughout the experiments to ensure equal food intake. Experiments were conducted in accordance with the regulations set forward by the national animal protection committee and in compliance with European Community Directive 2010/63. A local animal protection committee at SSI, IACUC, headed by DVM Kristin Engelhart Illigen, approved all experiments.

### Experimental models

Low-dose aerosol infection model: Female CB6F1 mice were infected by the aerosol route with ~25–50 CFU Mtb Erdman. Four weeks post challenge, mice were put on an 8-week course of normal chow (2916 Teklad Global 16% Protein Rodent Diet, Envigo) or chow containing 500 mg/kg celecoxib (LC Laboratories, Cat. No. C-1502) (Fig. 1 and Fig. 3). In one experiment (indicated in the text), mice were treated with oral ibuprofen (Nurofen orange flavor, purchased from pharmacy) in a daily dose of 80 mg/kg (2.24 mg/mice), which the mice was offered to eat from a pipette tip (Fig. 2).

High-dose aerosol infection model: Female CB6F1 mice were infected by the aerosol route with ~150–300 CFU Mtb Erdman. Three weeks post challenge, mice were treated with ibuprofen (Nurofen orange flavour, purchased from pharmacy) administered orally from a pipette tip in a daily dose of 80 mg/kg. The health status of individual mice was monitored according to a validated clinical scoring system (0–5) derived from the consolidation Act on Experimental Animals (BEK-88 30.01.2013) and mice were euthanized when they reached defined humane endpoints. The staff had several years of experience with the use of this scoring system: The mouse was unaffected (0), the mouse was slightly affected (slower movement and possibly slight bristle fur) (1), the mouse was obviously affected (immobile when inspected from outside the cage but mobile when the cage was moved, possible bristled fur, slightly changed breathing, eyes slightly closed, buckled back) (2), the mouse was moderately affected (mobile when physically touched, bristled fur, clearly changed breathing, eyes half closed, buckled back) (3), the mouse was heavily affected (almost immobile when physically touched, bristled fur, changed breathing, eyes closed) (4), the mouse was immobile and cold (5). The mice were euthanized when they reached a score of 2 or over.

Intravenous infection model: Female C3HeB/FeJ or CB6F1 mice were i.v. infected with 5.0 × 10^4^ CFU Mtb H37Rv or Mtb Erdman, respectively. Three weeks post infection, mice were treated with ibuprofen (Nurofen orange flavour, purchased from pharmacy) administered orally from a pipette tip in a daily dose of 80 mg/kg for 1 week (Fig. 2).

Re-infection model: Female CB6F1 mice were infected with ~25–50 CFU Mtb Erdman by the aerosol route. Four weeks after infection mice were fed with celecoxib chow for 8 weeks. Starting at 10 weeks post infection, mice were put on a course of isoniazid (0.1 g L^−1^) and rifabutin (0.1 g L^−1^) (Becton Dickinson) in the drinking water ad libitum for 12 weeks. After 2 weeks of rest, to wash out antibiotics, mice were re-challenged with ~50–100 CFU of Mtb Erdman 24 weeks post the first infection (Fig. 3).

Adoptive transfer model: 7- to 8week-old female C57BL/6J wild type mice were infected with ~25–50 CFU Mtb Erdman by the aerosol route. After 4 weeks of celecoxib-treatment, CD4 T-cells were purified from the spleen, tracheobronchial and mediastinal lymph nodes by negative selection (EasySep^TM^ Mouse CD4^+^ T cell Isolation Kit). A total of 9.5 × 10^6^ CD4 T cells in 200 µl from either C57BL/6J wild type mice or celecoxib-treated mice were transferred by i.v. injection to T cell receptor α-deficient B6.129S2-*Tcra*^tm1Mom^/J recipient mice that were infected 1 week earlier with Mtb Erdman. A control group did not receive any T cells (Fig. 4).

### Sample size

In the low-dose aerosol challenge model (incl. the adoptive transfer study), we powered the experiments for detecting a treatment effect 0.5 log10 CFU reduction in lungs compared to non-treated controls with a type I error rate of 5% (*α* = 0.05), a power of 80% and standard deviation of 0.35 log10 CFU (based on previous experiments). This resulted in *n* = 8 mice per group for primary analysis. We had no prior experience with the intravenous infection model and no power calculations were made. To ensure conclusive results, we increased the sample size to *n* = 13. Similarly, in the high-dose aerosol challenge model, a large sample size of *n* = 32 was selected due to the binary endpoint of dead/alive. In the re-infection model, we powered the experiment for detecting a treatment effect of 0.5 log10 CFU reduction compared to non-treated controls with a type I error rate of 5% (*α* = 0.05), a power of 80% and standard deviation of 0.5 log10 CFU. This resulted in *n* = 16 mice per group for primary analysis.

### Mtb infections and CFU enumeration

Mtb Erdman (TMC 107, ATCC) or Mtb H37Rv (ATCC) were grown to log phase in 7H9  medium enriched with Middlebrook ADC (BD Pharmingen, San Diego, CA, USA) without Tween. Log-phase bacteria were aliquoted and stored at −80 °C. For in vivo infection, vials of Mtb Erdman or H37Rv were thawed and sonicated for 5 min in a water bath. Furthermore, bacterial clumps were removed by dispersing the suspensions through a syringe.

For i.v. infections, mice were infected with 5.0 × 10^4^ CFU/mice in 100 µl injected into the tail vein. For aerosol infections, the inoculum was aerosolised for inhalation using a Biaera exposure system controlled via AeroMP software (Biaera, Hagerstown, MD, USA). The standard dose used in this study results in approximately 25–50 CFU/mouse.

The bacterial burden in lung was determined by homogenising the left lung lobe using M-tubes and a Miltenyi AutoMACS Dissociator (Miltenyi, Germany). The homogenate was serially diluted in 1x PBS containing panta (BD) and plated onto 7H11 MiddleBrook agar plates (BD). Colony forming units (CFU’s) were enumerated after 2 weeks of incubation at 37 °C, 5% CO_2_.

### Preparation of tissue and single cell suspension

Right lung lobes and spleens were aseptically removed from euthanatized mice and put into 2.5 mL cold RPMI. Lungs were mildly homogenized using T-tubes GentleMACS (Miltenyi Biotec), lung supernatants were harvested for cytokine analysis, right before digesting the lungs using collagenase IV (Sigma-Aldrich) for 30–60 min at 37 °C. Single cell suspensions of spleenocytes or lung cells were obtained by passing the suspension through 100 μm cell strainers. Lung and spleen cells were counted with an automatic NucleoCounter® (ChemoMetec). Blood samples were taken by cardiac puncture in tubes containing EDTA and plasma samples were harvested for cytokine analysis. Cells were washed twice in RPMI before direct staining for tetramers and surface markers or overnight stimulation for ICS analysis.

### Intravascular staining of T cells

Anti-mouse CD45.2 FITC (clone 104; BD) was diluted 1:50 in 1x PBS, 2.5 μg/mouse was given in 250 μl i.v. via the tail vein 3–6 min prior to organ harvest to distinguish parenchymal-localized (iv^−^) and vasculature-associated (iv^+^) T cells.

### Staining for flow cytometry

Prior to surface staining, cells were Fc receptor blocked with anti-CD16/32 either in combination with the tetramer stain or not. The ESAT-6 tetramer (I-Ab:ESAT-6_4-17_-PE) and the negative control (I-Ab:hCLIP-PE) were provided by the NIH tetramer facility (Atlanta, GA, USA). Cells were stained with tetramers for 30 min at 37 °C. For the analysis of innate cells, surface staining with the following markers: anti-CD3-Bv650, Ly-6G-PE, Ly-6C-FITC, CD11b conjugated to APC–Cy7 or PerCP–Cy5.5, CD11c-APC, CD86-PE-Cy7 and MHCII-PerCP–Cy5.5 was performed at 4 °C. For intracellular cytokine staining, a total of 1–2 × 10^6^ spleen or lung cells were stimulated in vitro for 1 h in RPMI + 10% FCS containing 1 μg/ml anti-CD28 and anti-CD49d with or without 5 μg of ESAT-6 pepmix followed by 5 h in the presence of 10 μg/ml brefeldin A (Sigma-Aldrich) at 37 °C. Cells were kept at 4 °C overnight. Following, cells were washed once in FACS buffer (1x PBS containing 1% FCS) and surface stained using anti-CD3-Bv650, anti-CD4-Bv510, anti-CD44-Bv786, anti-PD-1-Bv421 and anti-KLRG1-Bv711 at 4 °C for 15–30 min. Cells were washed in FACS buffer twice and permeabilized using the Cytofix/Cytoperm kit (BD) according to the manufacturer’s instructions. Following two washings steps, cells were stained intracellularly with anti–IFNγ PE-Cy7, anti–TNF–PE, anti-IL-2–APC–Cy7, anti-IL-17A–PerCP–Cy5.5 for 20–30 min. For transcription factor staining, the FoxP3/transcription factor buffer set (eBioscience) was used with anti-T-bet-ef660, RORγt-PE-CF594, FoxP3-FITC and GATA3-BV421. Cells were subsequently washed, resuspended in FACS buffer and analysed with a LSR FACS Fortessa (BD). Dead cells were excluded from the analysis by the fixable viability ef780. All flow cytometry data was analysed with FlowJo software v.10 (Tree Star, Ashland, OR, USA).

### Cytokine multiplex assay

The levels of pro-inflammatory cytokines in plasma samples and lung supernatants were evaluated using a multiplex Meso Scale Discovery assay (MSD). The pro-inflammatory Panel 1 (Mouse) 7-plex MSD assay measuring IL-1β, KC/GRO, IL-6, IL-12p70, IFNγ, TNFα, and IL-10 was performed according to the manufacturer’s instructions. A Sector Imager 2400 system (Meso Scale Discovery) was used for analysing the plates. The pro-inflammatory cytokine concentrations in unknown samples were calculated by a 4-parameter logistic non-linear regression analysis of the standard curve. Data on IL-10 is not shown as levels were below detection limit.

### Immunohistochemistry and iNOS staining

Right lobes were perfused with- and immersed in 4% phosphate-buffered formaldehyde (pH. 7.3) and stored at 4 °C. Lungs were further processed at BioSiteHisto (Helsinki, Finland). Each lobe was paraffin-embedded in blocks and sliced in four-millimeter thick sections using water fall rotation microtomes. Each sample was stained with haematoxylin and eosin. Inducible nitric oxide synthase (iNOS) was detected by immunohistochemistry using a polyclonal rabbit antibody in 1:200 dilution (Cat. # ab15323, BioSiteHisto). Briefly, paraffin sections were heated on a heating plate (+65 °C, 20 min.) to get better adherence between slide and section. Deparaffinisation was done by incubation in xylene for 10 min. After that, slides were moved into Leica Autostainer (Labvision) for additional paraffin removing, rehydration, and immunohistochemical staining, counterstaining, dehydration and mounting. Heat induced epitope retrieval was performed using a PT-module (Lab Vision) in TRIS-EDTA buffer pH9 at 95–100 °C. A 15 min heating was used to reach the correct temperature followed by 20 min epitope retrieval in the mentioned temperature. Changes in histopathology was assessed by a skilled pathologist that was blinded for both group association and treatment.

### Statistics and reproducibility

The effect of COXi treatment was assessed by an unpaired t-test between treated and non-treated animals unless otherwise indicated. Comparison of pro-inflammatory cytokines measured by multiplex was performed using an ANOVA followed by the Benjamini-Hochberg method to control the false discovery rate during multiple comparisons. For these comparisons we report the q values, which is the adjusted p value. A chi-square test was used to calculate the p value in the assessment of survival after high-dose aerosol challenge. A value of *p* < 0.05 was considered significant. All statistical analyses were carried out in GraphPad Prism version 7.04 (GraphPad Software Inc.).

### Blinding

The investigator was not involved in data collection. Organ homogenization, plating and CFU counting was performed by an experienced technician, who was not involved in study design and/or data analysis. Changes in histopathology was performed by a skilled pathologist who was blinded for both group association and treatment. The investigator was not blinded during data analysis and interpretation.

### Reporting summary

Further information on research design is available in the Nature Research Reporting Summary linked to this article.

### Supplementary information

Supplementary Information

Description of Additional Supplementary Files

Supplementary Data 1

Peer Review File

Reporting Summary

## Data Availability

The datasets that support the finding of this study are available from the corresponding author on reasonable request.
