# Author Correction: Cyclooxygenase inhibitors impair CD4 T cell immunity and exacerbate *Mycobacterium tuberculosis* infection in aerosol-challenged mice

**DOI:** 10.1038/s42003-024-06260-z

**Published:** 2024-05-29

**Authors:** Rasmus Mortensen, Helena Strand Clemmensen, Joshua S. Woodworth, Marie Louise Therkelsen, Tehmina Mustafa, Kristian Tonby, Synne Jenum, Else Marie Agger, Anne Ma Dyrhol-Riise, Peter Andersen

**Affiliations:** 1 Department of Infectious Disease Immunology, Statens Serum Institut, 2300 Copenhagen S, Denmark; 2 Centre for International Health, Department of Global Public Health and Primary Care, University of Bergen & Department of Thoracic Medicine, Haukeland University Hospital, 5021 Bergen, Norway; 3 Department of Infectious Diseases, Oslo University Hospital, 0424 Oslo, Norway; 4 Institute of Clinical Medicine, University of Oslo, 0424 Oslo, Norway; 5 Department of Clinical Science, University of Bergen, 5020 Bergen, Norway; 6 Department of Immunology and Microbiology, University of Copenhagen, 2200 Copenhagen N, Denmark

Correction to: *Communications Biology* 10.1038/s42003-019-0530-3, published online 05 August 2019

In the Methods section of this Article, there was an incorrect mention of a counting device as NucelocounterTM (Chemotec). This has now been corrected to NucleoCounter® (ChemoMetec).

