# Supplementary material for: Cyclooxygenase inhibitors impair CD4 T cell immunity and exacerbate Mycobacterium tuberculosis infection in aerosol-challenged mice
Source: Commun Biol. 2019 Aug 5;2:288. doi: 10.1038/s42003-019-0530-3 (PMC6683187; doi:10.1038/s42003-019-0530-3)

Supplementary Figure 1.
Example of gating strategy to identify innate cell populations in lungs of Mtb infected animals. In the “Singlets” gate, neutrophils were defined as CD11b^{+}/Ly-6C^{dim}/Ly-6G^{+} and with in the “not neutrophils” gate, inflammatory monocytes were identified as CD11b^{+}/Ly-6C^{high}. Gating on Ly-6C^{-}/Ly-6G^{-} CD11b^{+}cells, macrophages/monocytes (Mø/Mo) were defined as CD11b^{+}/MHCII^{+}/CD11c^{-} and a mixed population of alveolar macrophages/dendritic cells (AlvMø/DCs) was defined as CD11b^{+}/MHCII^{+}/CD11c^{+}. Expression of MHCII and CD86 (example shown) in AlvMØ/DCs was assessed by calculating the mean fluoresence intensity (MFI).

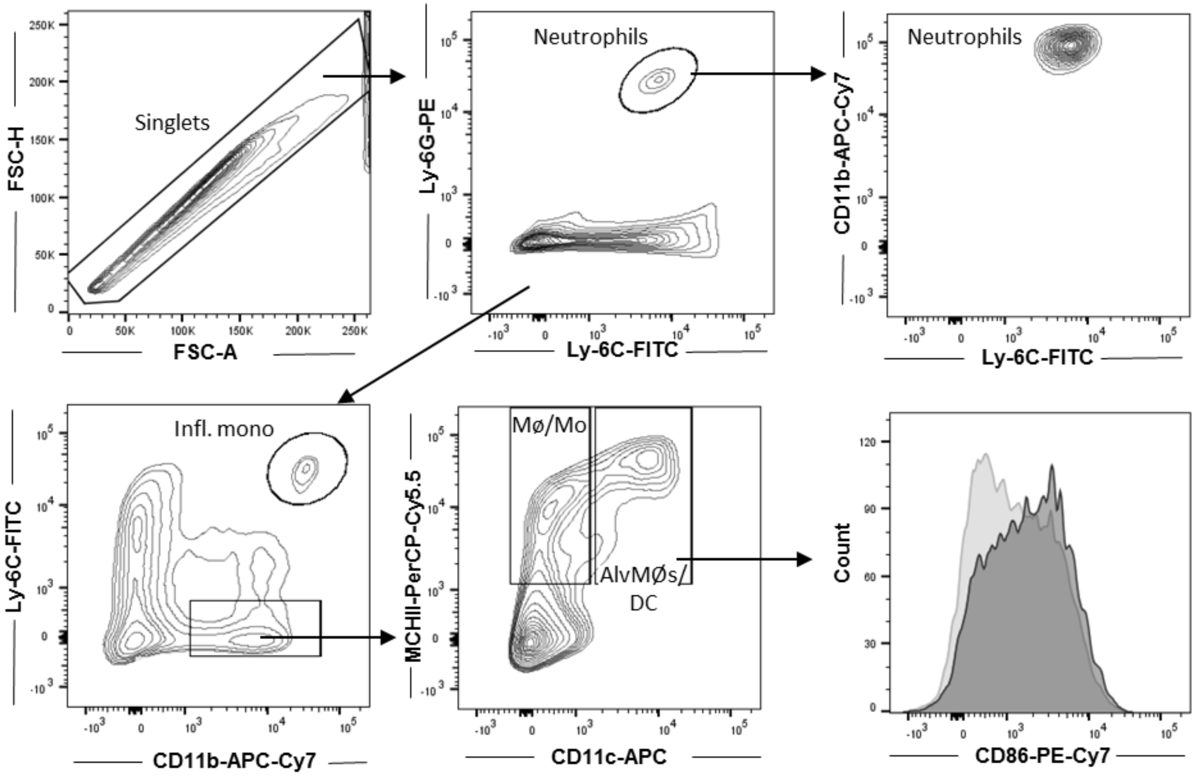

Supplementary Figure 2.
(A) Female CB6F1 mice of five to eight weeks of age were infected with ~25-50 CFU Mtb Erdman by the aerosol route. Four weeks into the infection, mice were fed with normal chow or chow containing 500ppm celecoxib. Two weeks after treatment (week 6 of the infection), the activation status of $CD11b^{+}CD11c^{+MHCII+}$ cells in the lung was assessed by staining for CD86 and MHCII (n=6). **(B,C)** Two weeks after treatment (week 6 of the infection) soluble proinflammatory cytokines were measured in plasma (B) or lung homogenates (C) by multiplex analysis (n=6). Bars indicate means ± SEM. p values were calculated with a students t-test and q values (adjusted p values) were calculated with an ANOVA followed by the Benjamini-Hochberg method to control the false discovery rate during multiple comparisons.

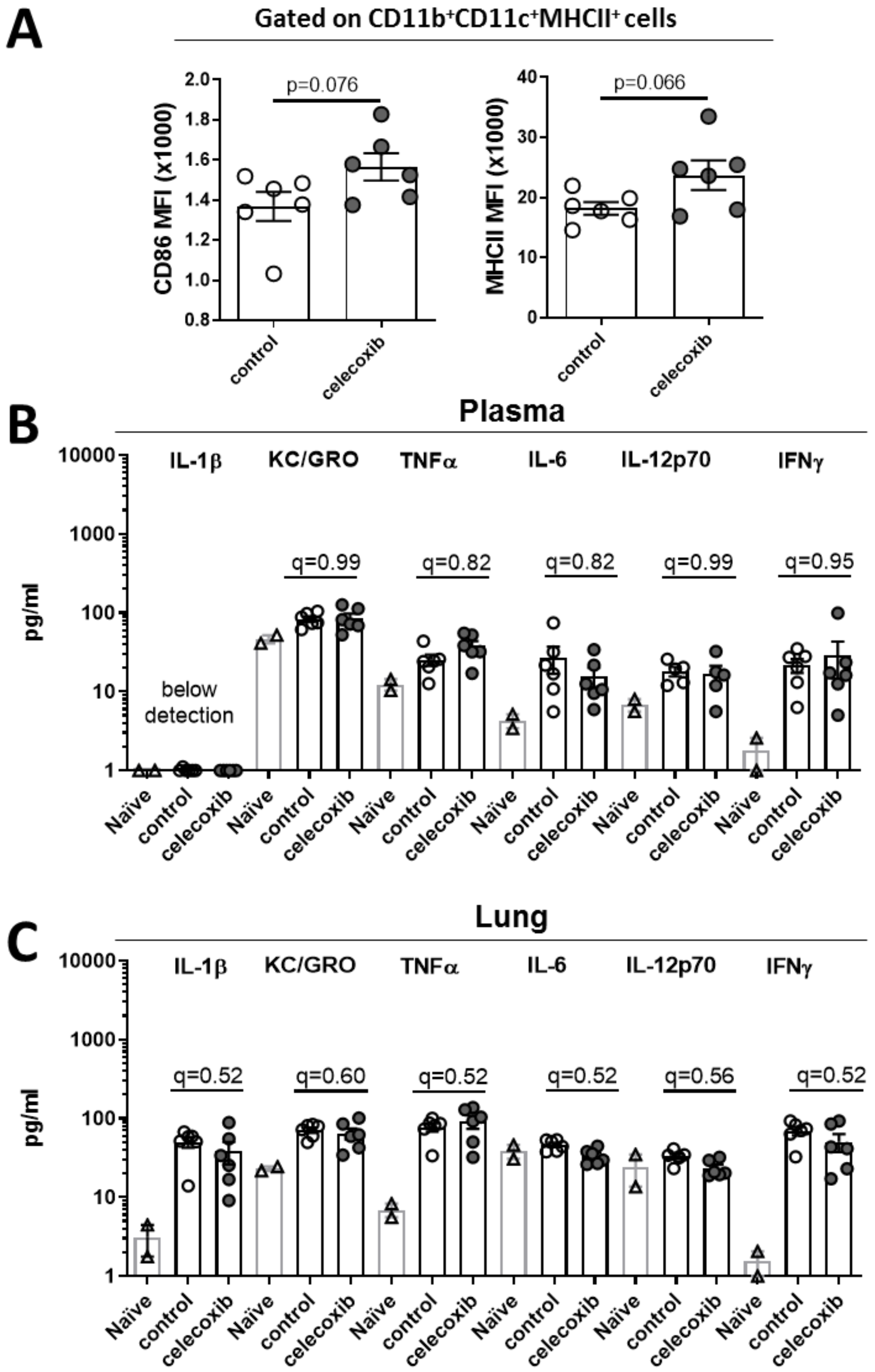

Supplementary Figure 3.
Soluble proinflammatory cytokines measured in lung homogenates of C3HeB/FeJ (n=4) by multiplex analysis one week of ibuprofen treatment (week four after i.v. infection). Bars indicate means ± SEM. q values (adjusted p values) were calculated with an ANOVA followed by the Benjamini-Hochberg method to control the false discovery rate during multiple comparisons.

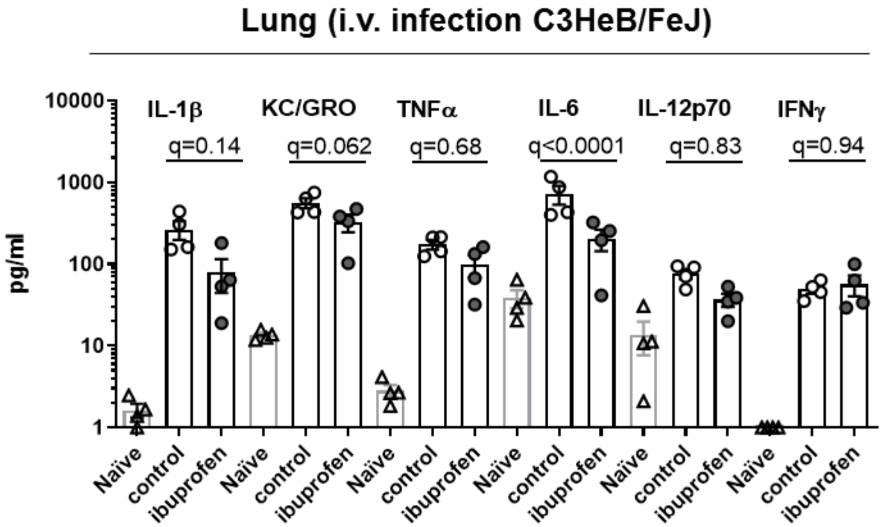

Supplementary Figure 4.

(A) CB6F1 mice were infected with ~25-50 CFU Mtb Erdman by the aerosol route, treated with celecoxib for eight weeks as previously and cleared with antibiotic treatment before Mtb reinfection (50-100 CFUs). Mtb bacilli were enumerated in the spleen by plating spleen homogenates at week six of the re-infection (n=16). **(B)** Lungs were harvested at week six (n=10) of the re-infection to investigate whether the initial celecoxib treatment induced changes in histopathology. **(left)** Hematoxylin and eosin stain of representative tissue sections of formalin fixed lungs. **(right)** percentage of lesion area (granulation lesion area/lung tissue area*100) for individual animals in each group. **(C)** Analysis of iNOS expression by immunohistochemistry (n=10). **(left)** Percentage of the iNOS$^+$ area within the total lesion area for individual animals in each group, **(middle)** iNOS staining of representative tissue sections of formalin fixed lungs, **(right)** correlation between lung bacterial burden and iNOS staining showing that higher lung CFU is associated with more iNOS staining in the lesions. **(D)** CB6F1 mice (n=6) were infected with ~25-50 CFU Mtb Erdman by the aerosol route and treated with celecoxib after four weeks. Two weeks into the treatment, the cytokine expression profile of lung CD4 T cells stimulated by overlapping ESAT-6 peptides was assed by ICS followed by combinatorial Boolean gating (see Supplementary Figure 5). Pies indicate the proportion of cell subsets expressing different combinations of cytokines: IFNγ$^+$ single positive (red), IFNγ$^+$TNF$^+$ double positive (orange) and IFNγ$^+$TNF$^+$IL-2$^+$ triple positive CD4 T cells (green). **(E)** Analysis of PD-1$^-$KLRG1$^+$ CD4 T cells producing any cytokine combination after stimulation with overlapping ESAT-6 peptides (n=6). Bars indicate means ± SEM. p values were calculated with a students t-test. In the correlation plot in Supplementary Figure 3C (right) a linear regression model was used to calculate the p value and r^2 coefficient.

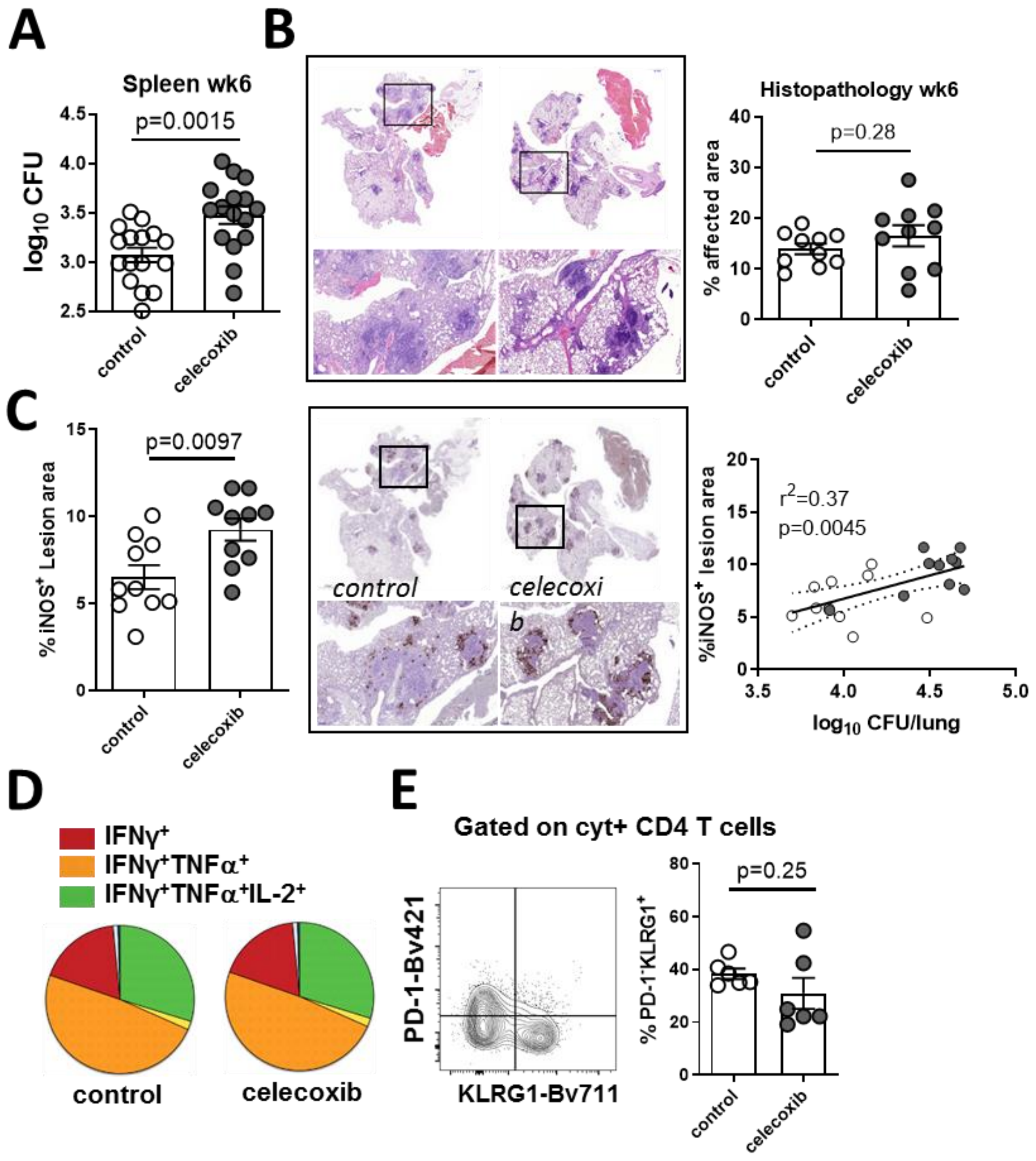

Supplementary Figure 5.
Example of gating strategy for intracellular cytokine staining (ICS) of stimulated CD4 T cells from Mtb infected lungs. In the “lymphocytes” gate, CD4 T cells were defined as $CD3^{+}CD4^{+}$. $CD44^{hi}$ Cytokine expressing cells, expressing any combination of IFNγ, TNFα or IL-2, were identified via Boolean gating. Parenchymal-localized T cells were identified by gating on cells that were not stained by the intravenously injected CD45-FITC antibody. Tbet expression was assessed in all CD4 T cells as wells as in cytokine producing CD4 T cells.

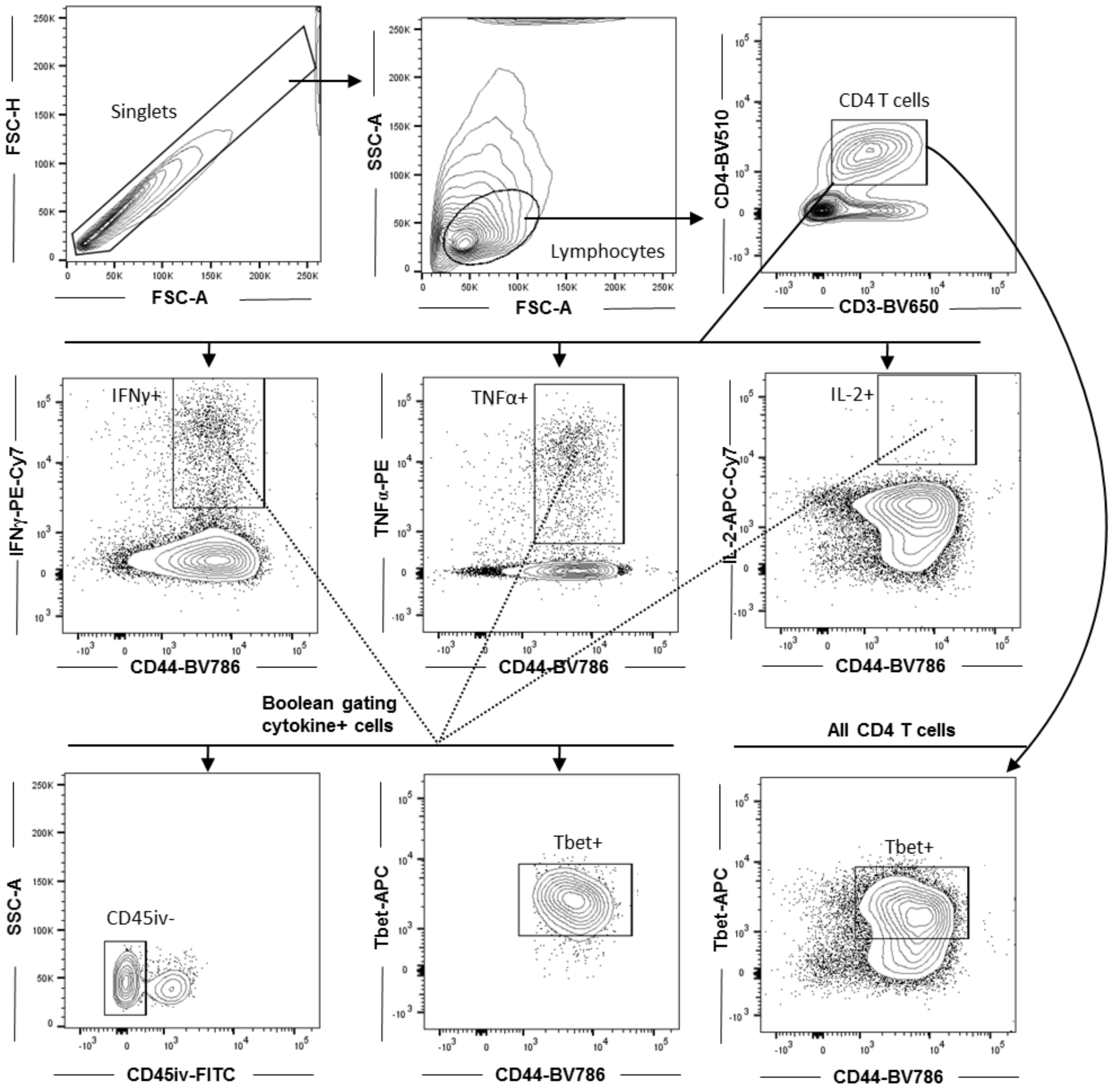

Supplementary Figure 6.
(A) Example of gating strategy to identify ESAT$_{4-17}$ tetramer positive CD4 T cells in pooled lymph nodes and spleens from Mtb infected animals. (**B**) Staining for FoxP3, RORγt and GATA-3 in ESAT-6$_{4-17}$ positive CD4 T cells purified from pooled spleen and LNs harvested from infected WT C57BL/6 mice four weeks into celecoxib treatment.

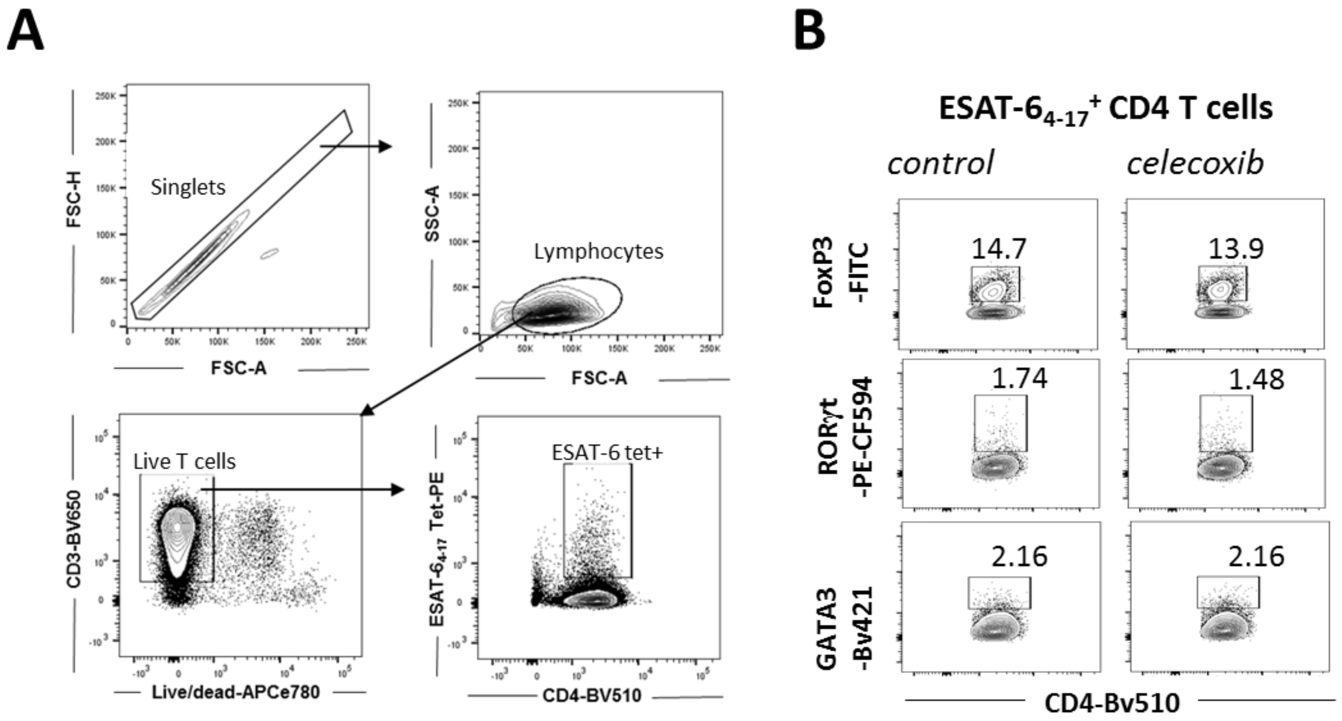

Supplement: Supplementary file 1 — Supplementary Information [file 42003_2019_530_MOESM1_ESM.pdf]
