# Supplementary material for: Cyclooxygenase inhibitors impair CD4 T cell immunity and exacerbate Mycobacterium tuberculosis infection in aerosol-challenged mice
Source: Commun Biol. 2019 Aug 5;2:288. doi: 10.1038/s42003-019-0530-3 (PMC6683187; doi:10.1038/s42003-019-0530-3)
Supplement: Supplementary file 2 — Description of Additional Supplementary Files [file 42003_2019_530_MOESM2_ESM.docx]

**Description of Additional Supplementary Files**

**File Name**: Supplementary Data 1

**Description**:  Source data for Figure 1-4
