# Supplementary material for: Cyclooxygenase inhibitors impair CD4 T cell immunity and exacerbate Mycobacterium tuberculosis infection in aerosol-challenged mice
Source: Commun Biol. 2019 Aug 5;2:288. doi: 10.1038/s42003-019-0530-3 (PMC6683187; doi:10.1038/s42003-019-0530-3)
Supplement: Supplementary file 5 — Reporting Summary [file 42003_2019_530_MOESM5_ESM.pdf]

nature research

Corresponding author(s): Rasmus Mortensen

Last updated by author(s): Jun 4, 2019

Reporting Summary

Nature Research wishes to improve the reproducibility of the work that we publish. This form provides structure for consistency and transparency in reporting. For further information on Nature Research policies, see Authors & Referees and the Editorial Policy Checklist.

Statistics

For all statistical analyses, confirm that the following items are present in the figure legend, table legend, main text, or Methods section.

n/a	Confirmed	
☐	☒	The exact sample size (n) for each experimental group/condition, given as a discrete number and unit of measurement
☐	☒	A statement on whether measurements were taken from distinct samples or whether the same sample was measured repeatedly
☐	☒	The statistical test(s) used AND whether they are one- or two-sided<br>*Only common tests should be described solely by name; describe more complex techniques in the Methods section.*
☒	☐	A description of all covariates tested
☐	☒	A description of any assumptions or corrections, such as tests of normality and adjustment for multiple comparisons
☐	☒	A full description of the statistical parameters including central tendency (e.g. means) or other basic estimates (e.g. regression coefficient) AND variation (e.g. standard deviation) or associated estimates of uncertainty (e.g. confidence intervals)
☐	☒	For null hypothesis testing, the test statistic (e.g. F, t, r) with confidence intervals, effect sizes, degrees of freedom and P value noted<br>*Give P values as exact values whenever suitable.*
☒	☐	For Bayesian analysis, information on the choice of priors and Markov chain Monte Carlo settings
☒	☐	For hierarchical and complex designs, identification of the appropriate level for tests and full reporting of outcomes
☒	☐	Estimates of effect sizes (e.g. Cohen's d, Pearson's r), indicating how they were calculated

Our web collection on statistics for biologists contains articles on many of the points above.

Software and code

Policy information about availability of computer code

Data collection	N/A
Data analysis	N/A

For manuscripts utilizing custom algorithms or software that are central to the research but not yet described in published literature, software must be made available to editors/reviewers. We strongly encourage code deposition in a community repository (e.g. GitHub). See the Nature Research guidelines for submitting code & software for further information.

Data

Policy information about availability of data

All manuscripts must include a data availability statement. This statement should provide the following information, where applicable:

- Accession codes, unique identifiers, or web links for publicly available datasets
- A list of figures that have associated raw data
- A description of any restrictions on data availability

The datasets that support the finding of this study are available from the corresponding author on reasonable request.

Field-specific reporting

Please select the one below that is the best fit for your research. If you are not sure, read the appropriate sections before making your selection.

☒ Life sciences ☐ Behavioural & social sciences ☐ Ecological, evolutionary & environmental sciences

For a reference copy of the document with all sections, see nature.com/documents/nr-reporting-summary-flat.pdf

Life sciences study design

All studies must disclose on these points even when the disclosure is negative.

Sample size	In the standardized murine aerosol challenge model (incl. the adoptive transfer study), we powered the experiments for detecting a treatment effect 0.5 log10 CFU reduction in lungs compared to non-treated controls with a type I error rate of 5% (α=0.05), a power of 80% and standard deviation of 0.35 log10 CFU (based on previous experiments). This results in n=8 mice per group for primary analysis. We had no prior experience with the intravenous infection model and no power calculations were made. To ensure conclusive results, we increased the samplesize to n=13. The same goes for the high-dose challenge study where n=32 was selected due to the binary endpoint of "dead/alive". In the re-infection model, we powered the experiment for detecting a treatment effect of 0.5 log10 CFU reduction compared to non-treated controls with a type I error rate of 5% (α=0.05), a power of 80% and standard deviation of 0.5 log10 CFU. This results in n=16 mice per group for primary analysis.
Data exclusions	No data points was excluded from the analysis
Replication	The main finding of this paper, is that COXi treatment increase the bacterial burden after Mtb aerosol challenge in mice. We demonstrate this effect in Five different experimental setups (a total of six experiments): 1. celecoxib treatment in the stadardized aerosol infection model (one experiment), 2. ibuprofen (different COXi) treatment in the stardized aerosol infection model (two experiments),3 . Ibuprofen treatment during lethal high-dose aerosol infection 4. celecoxib treatment in a re-infection model (one experiment) and 5. adoptive transfer of CD4 T cells from celecoxib treated mice (one experiment). Our appraoch of using five different experimental set-ups (reflecting four different aspects of the treatment effect) ensures robustness of our findings. Since our results contradicted previous published studies, we validated that we could replicate their data when we adapted their experimental approach (two experiments with intravenous infection).
Randomization	Mice were randomly assigned to cages upon arrival to our animal facility. For each time point in the study, mice were randomly selected for primary analysis.
Blinding	The investigator was not involved in data collection. Organ homogenization, plating and CFU counting was performed by an experienced technician, who was not involved in study design and/or data analysis. Changes in histopathology was performed by a skilled pathologist who was blinded for both group association and treatment. The investigator was not blinded during data analysis and interpretation.

Reporting for specific materials, systems and methods

We require information from authors about some types of materials, experimental systems and methods used in many studies. Here, indicate whether each material, system or method listed is relevant to your study. If you are not sure if a list item applies to your research, read the appropriate section before selecting a response.

Materials & experimental systems

n/a	Involved in the study	
☐	☒	Antibodies
☒	☐	Eukaryotic cell lines
☒	☐	Palaeontology
☐	☒	Animals and other organisms
☒	☐	Human research participants
☒	☐	Clinical data

Methods

n/a	Involved in the study	
☒	☐	ChIP-seq
☐	☒	Flow cytometry
☒	☐	MRI-based neuroimaging

Antibodies

Antibodies used	CD45.2-FITC (BD Bioscience, clone: 104, lot: 6134772), CD44-FITC (eBioscience, clone: IM7, lot: 43122478), CD44-Bv786 (BD Bioscience, clone: IM7, lot: 7158677), CD3-BV650 (Biolegend, clone: 17A2, lot: B261371), CD4-Bv510 (Biolegend, clone: RM4-5, lot: B252816), Viability dye-ef780 (eBioscience, clone: n/a, lot: 1949295), KLRG1-Bv711 (Biolegend, clone: 2F1, lot: B250417), PD-1-Bv421 (Biolegend, clone: 29F.1A12, lot: B231474), T-bet-APC (Invitrogen, clone: eBio4Bio, lot: E113177), IFN-y-PE-Cy7 (Invitrogen, clone: XMG1.2, lot: 4332567), TNF-a-PE (eBioscience, clone: MP6-XT22, lot: 4318513), IL-2-APC-Cy7 (BD Bioscience, clone: JES6-5H4, lot: 8275523), IL-17-PerCpCy5.5 (eBioscience, clone: XMG1.2, lot: 4289632), Ly6G-PE (BD Bioscience, clone: IA8, lot: 7240979), Ly6C-FITC (BD Bioscience, clone: AL-21, lot: 33380), CD11b-APC-Cy7 (Biolegend, clone: M1/70, lot: B2113161), CD11b-PerCpCy5.5 (BD Bioscience, clone: M1/70, lot: 7066555), CD11c-APC (BD Bioscience, clone: HL3, lot: 5016523), MHC-II-PerCp-Cy5.5 (Biolegend, clone: M5/114.15.2, lot: B253463), CD28 (BD Bioscience, clone: 37.51, lot: 8086754), CD49 (BD Bioscience, clone: 9C10(MFR4B), lot: 8149619), Fc-block (BD Bioscience, clone: 2.462, lot: 7157841), FoxP3-FITC (eBioscience, clone: FJK-16s, lot: 4340671), GATA3-BV421 (BD Bioscience, clone: L50-823, lot: 8256547), RORγt-PE-CF594 (BD Bioscience, clone: Q31-378, lot: 8298705).
Validation	Validation in the target species was performed by the supplier of the antibody.

Animals and other organisms

Policy information about studies involving animals; ARRIVE guidelines recommended for reporting animal research

Laboratory animals	C3HeB/FeJ (The 'Jackson' Laboratories, Bar Harbor, ME), CB6F1 hybrid mice (female BALB/c x male C57BL/6, 'Envigo' Laboratories, The Netherlands), C57BL/6 (Charles River Laboratories, Wilmington, MA) and B6.129S2-Tcratm1Mom/J (Jackson). All mice were females, 5-8 weeks old when the experiments were initiated and kept in cages of 8 animals in total. Animals had access to ad libitum chow and drinking water during the experiments.
Wild animals	Not relevant.
Field-collected samples	Not relevant.
Ethics oversight	Experiments were conducted in accordance with the regulations set forward by the Danish Ministry of Justice and animal protection committees by Danish Animal Experiments Inspectorate Permit 2004-561-868 (of January 7, 2004) and in compliance with European Community Directive 2010/63/EU of the European parliament and of the council of 22 September 2010 on the protection of animals used for scientific purposes, as well as Directive 86/609 and the U.S. Association for Laboratory Animal Care recommendations for the care and use of laboratory animals. The experiments were approved by a local animal protection committee at Statens Serum Institut, IACUC, headed by DVM Kristin Engelhart Illigen.

Note that full information on the approval of the study protocol must also be provided in the manuscript.

Flow Cytometry

Plots

Confirm that:

☒ The axis labels state the marker and fluorochrome used (e.g. CD4-FITC).

☒ The axis scales are clearly visible. Include numbers along axes only for bottom left plot of group (a 'group' is an analysis of identical markers).

☒ All plots are contour plots with outliers or pseudocolor plots.

☒ A numerical value for number of cells or percentage (with statistics) is provided.

Methodology

Sample preparation	Tissue samples were prepared by mechanically forcing the tissue through a 100 um cell strainer. For lung samples, a pre-processing step were necessary. Lungs were grained using T-tubes (Miltenyi Biotec) and treated with collagenase for 30-60 minutes at 37 degrees, 5% CO2. Cells were washed twice in RPMI and finaly resuspended in RPMI + 10% FCS. Cells were at this point on single cell level and ready for staining analysis.
Instrument	Samles were analyzed with a BD LSRFortessa™ using a BD™ High Throughput Sampler (HTS).
Software	Data management was carried out with BD FACSDiva™ v6.2 software. Data was analyzed using FlowJo software v.10 (Tree Star, Ashland, OR, USA).
Cell population abundance	No cell sorting was performed with FACS.
Gating strategy	For T-cell analyses, cells were gating for singlets (FSC-A/FSC-H), lymphocytes (FSC-A/SSC-A) and a T cell gate (CD3-Bv650/CD4-Bv510). Subsequently, cells were either gated for tetramer specific T cells (I-Ab ESAT-64-17-PE) or cytokine producing T cells (CD44-FITC/IFNγ PE-Cy7, TNF–PE, L-2–APC–Cy7, IL-17A–PerCP–Cy5.5). Antigen-specific T cells were afterwards characterized for their expression of T-bet-APC, GATA3-BV421, RORγt-PE-CF594, FoxP3-FITC, PD-1-Bv421 or KLRG1-Bv711. Viability dye (viability-ef780) was used in the tetramer panels.<br><br>Myeloid-derived cells were gated on singlets and a CD3-Bv650 dump gate to exclude any T cells. Subset of innate cells were determined by staining lung cells for CD11b-APC-Cy7/CD11b-PerCp-Cy5.5, CD11c-APC, Ly-6C-FITC, Ly-6G-PE and MCHII-PerCp-Cy5.5, CD86-PE-Cy7. Neutrophils were defined as CD11b+/Ly-6Cdim/Ly-6G+, inflammatory monocytes as CD11b+/Ly-6Chi/Ly-6G-. Gating on Ly-6C-/Ly-6G- cells, macrophages/monocytes (Mø/Mo) were defined as CD11b+/MHCII+/CD11c- and a mixed population of alveolar macrophages/dendritic cells (Alv Mø/DCs) was defined as CD11b+/MHCII+/CD11c+.

☒ Tick this box to confirm that a figure exemplifying the gating strategy is provided in the Supplementary Information.
