# Supplementary material for: Cyclooxygenase inhibitors impair CD4 T cell immunity and exacerbate Mycobacterium tuberculosis infection in aerosol-challenged mice
Source: Commun Biol. 2019 Aug 5;2:288. doi: 10.1038/s42003-019-0530-3 (PMC6683187; doi:10.1038/s42003-019-0530-3)
Supplement: Supplementary file 4 — Peer Review File [file 42003_2019_530_MOESM4_ESM.pdf]

Editorial Note: This manuscript has been previously reviewed at another journal. This document only contains reviewer comments and rebuttal letters for versions considered at *Communications Biology.*

REVIEWERS' COMMENTS:

Reviewer #1 (Remarks to the Author):

This study demonstrates that oral NSAID therapy exacerbated aerosol infection with Mt reproducibly in the CB6Fi strain, and this effect was associated with reduced Th1 T cell differentiation. The authors have responded to the issues raised by the previous reviewers. In particular they have provided new data to show:
1. that NSAID therapy was associated with reduced survival after high do=se aerosol infection in the same mouse strain.
2. that there was no significant effect on the activation of lung DCs,
3. there was a non-statistically significant trend for increased pathology in the lungs, but make the correct point that this may relate to the increased bacterial load.
4. and provided adequate explanations for technical questions.

Therefore the data are robust and appropriately analysed.
Importantly they have modified their conclusions to clarify that the effect of NSAIDs varies in different models of murine TB, and the final conclusions of their effects in human TB will require careful analysis of ongoing clinical trials.
